# Effect of Soluble Epoxide Hydrolase on the Modulation of Coronary Reactive Hyperemia: Role of Oxylipins and PPARγ

**DOI:** 10.1371/journal.pone.0162147

**Published:** 2016-09-01

**Authors:** Ahmad Hanif, Matthew L. Edin, Darryl C. Zeldin, Christophe Morisseau, Mohammed A. Nayeem

**Affiliations:** 1 Basic Pharmaceutical Sciences, School of Pharmacy, Center for Basic and Translational Stroke Research, West Virginia University, Morgantown, WV, United States of America; 2 Division of Intramural Research, NIEHS/NIH, Research Triangle Park, NC, United States of America; 3 University of California at Davis, One Shields Avenue, Davis, CA, United States of America; Universita degli Studi di Catania, ITALY

## Abstract

Coronary reactive hyperemia (CRH) is a physiological response to ischemic insult that prevents the potential harm associated with an interruption of blood supply. The relationship between the pharmacologic inhibition of soluble epoxide hydrolase (sEH) and CRH response to a brief ischemia is not known. sEH is involved in the main catabolic pathway of epoxyeicosatrienoic acids (EETs), which are converted into dihydroxyeicosatrienoic acids (DHETs). EETs protect against ischemia/reperfusion injury and have numerous beneficial physiological effects. We hypothesized that inhibition of sEH by *t*-AUCB enhances CRH in isolated mouse hearts through changing the oxylipin profiles, including an increase in EETs/DHETs ratio. Compared to controls, *t*-AUCB–treated mice had increased CRH, including repayment volume (RV), repayment duration, and repayment/debt ratio (p < 0.05). Treatment with *t*-AUCB significantly changed oxylipin profiles, including an increase in EET/DHET ratio, increase in EpOME/DiHOME ratio, increase in the levels of HODEs, decrease in the levels of mid-chain HETEs, and decrease in prostanoids (p < 0.05). Treatment with MS-PPOH (CYP epoxygenase inhibitor) reduced CRH, including RV (p < 0.05). Involvement of PPARγ in the modulation of CRH was demonstrated using a PPARγ-antagonist (T0070907) and a PPARγ-agonist (rosiglitazone). T0070907 reduced CRH (p < 0.05), whereas rosiglitazone enhanced CRH (p < 0.05) in isolated mouse hearts compared to the non-treated. These data demonstrate that sEH inhibition enhances, whereas CYP epoxygenases-inhibition attenuates CRH, PPARγ mediate CRH downstream of the CYP epoxygenases-EET pathway, and the changes in oxylipin profiles associated with sEH-inhibition collectively contributed to the enhanced CRH.

## Introduction

Cytochrome P450 (CYP) epoxygenases catalyze the epoxidation of arachidonic acid (AA) to form epoxyeicosatrienoic acids (EETs). Produced in endothelial cells, EETs have numerous biological functions, including hyperpolarization and relaxation of vascular smooth muscle cells by activating large conductance Ca^2+^-activated K^+^ channels (BK_Ca_) [1, 2]. EETs are rapidly metabolized through hydration by soluble epoxide hydrolase (sEH) to the less active dihydroxyeicosatrienoic acids (DHETs). Although hydration by sEH is the main catabolic pathway of EETs [3], two EET isomers, 5,6- and 8,9-EET, are substrates for the cyclooxygenase (COX) pathway [4]. Different strategies can increase the levels of EETs to study their effects, including transgenic models [5–7] and pharmacologic targeting of sEH [8] or CYP epoxygenases [9]. EET generation was increased in CYP2J2–over-expressed mouse cardiomyocytes and was shown to exert cardioprotective effects against ischemia/reperfusion injury [5]. In addition to EETs, targeting sEH impacts the levels of oxylipins (epoxyoctadecaenoic acids [EpOMEs], dihydroxyoctadecaenoic acids [DiHOMEs], hydroxyoctadecadienoic acids [HODEs], hydroxyeicosatetraenoic acids [HETEs], and prostanoids) [10–13]. Lee et al. reported that sEH inhibition by AUDA was associated with an increased EpOME/DiHOME ratio and improved renal recovery against ischemia/reperfusion injury in C57BL/6 mice [14]. 13-HODE enhanced prostacyclin (PGI_2_) biosynthesis and was involved in splenic and coronary artery relaxation in Mongrel dogs [15]. Also, mid-chain HETEs had direct chemotactic effects on vascular tone and on the production of endothelial vascular factors [16–19]. Further, studies indicated that EET biological effects could be mediated by peroxisome proliferator-activated receptor-gamma (PPARγ) [20–23]. For example, EET-induced aortic relaxation in mice was mediated by PPARγ [7, 21].

When the heart is subjected to a brief period of ischemia, it responds by momentarily increasing coronary blood flow [24], known as reactive hyperemia (RH) or coronary RH (CRH). The phenomenon of CRH is associated with significant coronary vasodilation in response to a cessation in coronary perfusion. Ischemic insult is associated with potentially detrimental effects on cardiac function. Increased blood flow associated with CRH prevents or decreases injury or damage due to ischemia. By supplying an increased amount of blood to the deprived heart muscle, CRH speeds up functional recovery and washes away accumulated metabolic byproducts [25]. CRH is considered a protective mechanism, which is blunted or compromised in pathologic conditions affecting the coronary circulation, including cardiac hypertrophy [26], metabolic syndrome [27], unstable angina, myocardial infarction, and congestive heart failure [28]. The metabolic mediators involved in RH have been studied extensively, and include adenosine [26, 29, 30], nitric oxide (NO) [26], K_ATP_ channels [26] and hydrogen peroxide (H_2_O_2_) [26, 31].

The potential effects of pharmacologic inhibition of CYP epoxygenases, inhibition of sEH, and associated changes in oxylipin profiles on CRH in response to a short period of ischemia have not been investigated. We hypothesized that inhibition of sEH enhances CRH through modulation in oxylipin profiles and PPARγ, whereas inhibition of CYP epoxygenases attenuates CRH in isolated mouse hearts.

## Materials and Methods

### Animals

The generation of sEH null (sEH^–/–^) mice was described by Sinal et al. [3]. sEH^–/–^and wildtype (WT, sEH^+/+^) mice were of the C57BL/6 genetic background and provided by Dr. Darryl Zeldin, National Institute of Environmental Health Sciences/National Institutes of Health (NIH). All animal care and experimentation protocols were submitted to, approved, and carried out in accordance with the West Virginia University Institutional Animal Care and Use Committee and were in accordance with the principles and guidelines of the NIH’s *Guide for the Care and Use of Laboratory Animals*. Both male and female mice (14–16 wks old) in equal ratio were used in our study. Mice were maintained in cages with a 12:12 h light-dark cycle and free access to standard chow and water.

### Langendorff-Perfused Heart Preparation

There are two available Langendorff technique modes to perfuse the heart: 1) constant pressure mode and 2) constant flow mode. In our experiments, we selected the constant pressure mode. Because the two modes have important differences, the selection between the two is particularly important when assessing the role of coronary regulatory mechanisms in response to changing coronary flow (CF) conditions or pathologies, such as ischemia. The constant pressure mode is physiologically more relevant in experiments involving ischemia, like the model in our experiments; therefore, it was selected over the other mode [32].

Soluble epoxide hydrolase null (sEH^–/–^) and wild-type (WT; sEH^+/+^) mice (14–16 wks.) of both sexes (equal ratios) were euthanized with sodium pentobarbital (100 mg/kg body weight intra-peritoneally). Hearts were excised and immediately placed into heparinized (5 U/mL) ice-cold Krebs-Henseleit buffer containing (in mM) 119.0 NaCl, 11.0 glucose, 22.0 NaHCO_3_, 4.7 KCl, 1.2 KH_2_PO_4_, 1.2 MgSO_4_, 2.5 CaCl_2_, 2.0 pyruvate, and 0.5 EDTA. After removal of the lungs and tissue surrounding the heart, the aorta was rapidly cannulated with a 20-gauge, blunt-ended needle and continuously perfused with 37°C buffer continuously bubbled with [95% O_2_]–[5% CO_2_] at a constant perfusion pressure of 80 mmHg. The left atrium was excised, and a water-filled balloon made of plastic wrap was inserted into the left ventricle through the mitral valve. The balloon was connected to a pressure transducer for continuous measurement of left ventricular developed pressure (LVDP) and heart rate (HR). The heart was then immersed in a water-jacketed perfusate bath (37°C) and left to beat spontaneously. Left ventricular diastolic pressure was adjusted to 2–5 mmHg. A flow transducer was installed above the cannulated aorta for continuous measurement of CF with an ultrasonic flow probe (Transonic Systems, Ithaca, NY). A Power–Lab Chart data acquisition system (AD Instruments, Colorado Springs, CO) was used for data acquisition. Heart function was allowed to stabilize for 30–40 min before initiation of CRH. Only hearts whose CF increased by more than two fold after a 15-second total occlusion were included in the analysis. This demonstrated that the isolated heart preparation was intact to be included in the experiment. Hearts with persistent arrhythmias or LVDP <80 mmHg were excluded.

### Coronary Reactive Hyperemic Response

After stabilization for 30–40 minutes, baseline CF, HR, and LVDP were recorded. Hearts were subjected to 15 seconds of total occlusion by closing the valve directly above the cannulated heart to bring forth CRH. After CF returned to pre-CRH baseline levels, post-CRH baseline CF, CF tracing, peak hyperemic flow (PHF), HR, LVDP, repayment volume (RV), and repayment duration (RD) recordings were analyzed for each isolated heart. Investigational drugs were infused into the aortic perfusion line using a microinjection pump (Harvard Apparatus, Holliston, MA) for 15 minutes, after which another CRH was induced and the same parameters analyzed again. Drugs were infused at a rate equivalent to 1% of CF. The final concentrations, after standardization of dose (0.01, 0.1, 1, & 10 μM) response for the various drugs used in this study were 10 μM for T0070907 (PPARγ-antagonist), rosiglitazone (PPARγ-agonist), *t*-AUCB (trans-4-[4-(3-adamantan-1-yl-ureido)-cyclohexyloxy]-benzoic acid (a selective sEH-inhibitor, University of California, Davis), and 1 μM for MS-PPOH (methylsulfonyl-propargyloxyphenylhexanamide, CYP-epoxygenases inhibitor). These concentrations are the same, or less, as used in previous studies: rosiglitazone, 10 μM; [33], *t*-AUCB, 10 μM; [34], MS-PPOH, 1 μM [35].

### Effect of *t*-AUCB on CRH Response

After stabilization for 30–40 min, isolated WT mouse hearts were subjected to 15 sec of total occlusion. Recordings of the first CRH (baseline CF, CF tracing, LVDP, HR, RV, PHF, and RD) were analyzed for each heart and averaged. *t*-AUCB was infused at a final concentration of 10 μM and 1% of CF rate for 15 min, after which another CRH was induced and the same parameters (baseline CF, CF tracing, LVDP, HR, RV, and RD) were recorded again and analyzed.

### Effect of *t*-AUCB on CRH Response in sEH^–/–^Mice:

To investigate the specificity of the sEH-inhibitor, *t*-AUCB, we evaluated its effect on CRH in sEH^–/–^mouse hearts, where the sEH gene is globally deleted. We followed the same protocol described above. CRH was induced in sEH^–/–^mice before and after infusing *t*-AUCB for 15 min. The two CRH responses were analyzed.

### LC–MS/MS Oxylipin Analysis

Levels of oxylipins (5,6-, 8,9-, 11,12- and 14,15-EET, 5,6-, 8,9-, 11,12- and 14,15-DHET, 5-, 8-, 9-, 11-, 12- and 15-HETE, 9,10- and 12,13-EpOME, 9,10- and 12,13-DiHOME, 9- and 13-HODE, 6-keto prostaglandin-F_1α_ [6K-PG-F_1α_], PG-F_2α_, thromboxane B_2_ [TxB_2_], PGD_2_, and PGE_2_) were determined in pre- and post-CRH heart perfusates of WT and *t*-AUCB-treated WT mice through liquid chromatography, tandem mass spectroscopy (LC-MS/MS) as described previously [36]. Heart perfusates were collected after the first 30 min of stabilization and right after reperfusion for 2.5 min. Hearts were immersed in 5 mL of warm (37°C) Krebs-Henseleit buffer with 5 μL of 10 μM *t*-AUCB to block further EET breakdown by sEH. Heart perfusates were collected two times before ischemia (baseline) and pooled together as one sample and two times after ischemia and pooled together as another sample for LC-MS/MS analysis. Samples were stored at –80°C until processing. Samples were spiked with 30 ng PGE2-d4, 10,11- DiHN, and 10,11-EpHep (Cayman) as internal standards, mixed with 0.1 vol of 1% acetic acid in 50% methanol, and extracted by serial passage through Oasis HLB C18 3mL columns (Waters, Milford, MA, USA). Columns were washed twice with 0.1% acetic acid in 5% methanol and eluted with methanol into glass tubes containing 6 μL of 30% glycerol in methanol. The methanol was then evaporated under a stream of nitrogen gas, and the dried tubes were frozen and stored at –80°C until analysis. Online liquid chromatography of extracted samples was performed with an Agilent 1200 Series capillary HPLC (Agilent Technologies, Santa Clara, CA, USA). Separations were achieved using a Halo C18 column (2.7 mm, 10062.1 mm; MAC-MOD Analytical, Chadds Ford, PA), which was held at 50°C. Mobile phase A was 85:15:0.1 water: acetonitrile: acetic acid. Mobile phase B was 70:30:0.1 acetonitrile: methanol: acetic acid. Flow rate was 400 μL/min; Gradient elution was used. Mobile phase percentage B and flow rate were varied as follows: 20% B at 0 min, ramp from 0 to 5 min to 40% B, ramp from 5 to 7 min to 55% B, ramp from 7 to 13 min to 64% B. From 13 to 19 min the column was flushed with 100% B at a flow rate of 550 μL/min. Samples were solvated in 50 μl of 30% ethanol. The injection volume was 10 μL. Samples were analyzed in triplicate. Analyses were performed on an MDS Sciex API 3000 equipped with a TurboIonSpray source (Applied Biosystems). Turbo desolvation gas was heated to 425°C at a flow rate of 6 L/min. Negative ion electrospray ionization tandem mass spectrometry with multiple reaction monitoring was used for detection.

### Effect of MS-PPOH (CYP epoxygenase inhibitor) on CRH Response

Isolated WT mice hearts were stabilized for 30–40 min, followed by 15 sec of total occlusion. Recordings of the first CRH (baseline CF, CF tracing, LVDP, HR, RV, PHF and RD) were analyzed for each heart and averaged. MS-PPOH was infused at a final concentration of 1.0 μM for 15 minutes, after which the second CRH was induced. CRHs before and after MS-PPOH infusion were analyzed and compared.

### Effect of T0070907 (PPARγ antagonist) on CRH Response

Isolated WT mouse hearts were stabilized for 30–40 min followed by 15 sec of total occlusion. Recordings of the first CRH (baseline CF, CF tracing, LVDP, HR, RV, PHF and RD) were analyzed for each heart and averaged for each group, as mentioned previously. T0070907 was infused at a final concentration of 10 μM and 1% of CF rate for 15 min, after which another CRH was induced and the same parameters (baseline CF, CF tracing, LVDP, HR, RV, and RD) were recorded again and analyzed.

### Effect of *t*-AUCB and T0070907 on CRH Response

In this experiment, we investigated a possible link between the role of PPARγ in CRH and pharmacological inhibition of sEH by *t*-AUCB. We evaluated the effects of selective sEH-inhibition on CRH followed by PPARγ-antagonist. Baseline CRH was induced in WT mice. *t*-AUCB was infused at a final concentration of 10 μM for 15 minutes, after which a second CRH was induced. T0070907 was infused while *t*-AUCB was still being infused, at a final concentration of 10 μM for another 15 min, and a third CRH was induced. The three CRH responses were analyzed and compared.

### Effect of Rosiglitazone on CRH Response

After stabilization, WT mouse hearts were subjected to 15 sec of total occlusion. As described above, baseline CRH was induced in each mouse heart. Rosiglitazone was infused at a final concentration of 10 μM for 15 min, followed by another CRH. CRHs before and after rosiglitazone infusion were analyzed and compared.

### Statistical and Data Analyses

Flow debt (baseline flow rate multiplied by occlusion duration) and RV (the integral of hyperemic area above the baseline flow) were calculated using “the integral relative to baseline” function in the data pad of Lab-Chart 7.0 software. Since absolute coronary flow rates change proportionally with heart mass, the RV and flow debt are presented as ml/g wet heart weight, and baseline and peak flow rate data are presented as (mL.min^–1^.g wet heart weight^–1^). Values are means ± standard error; *n* represents the number of animals. For data analysis, two-tailed paired *t*-tests were used for paired data analysis, repeated measures ANOVAs were used for populations measured 3 times, and two-way ANOVAs were used to compare data between groups. Differences were considered statistically significant when p < 0.05.

## Results

### CRH Response

#### Effect of *t*-AUCB on CRH Response in WT Mice

**Fig 1:**
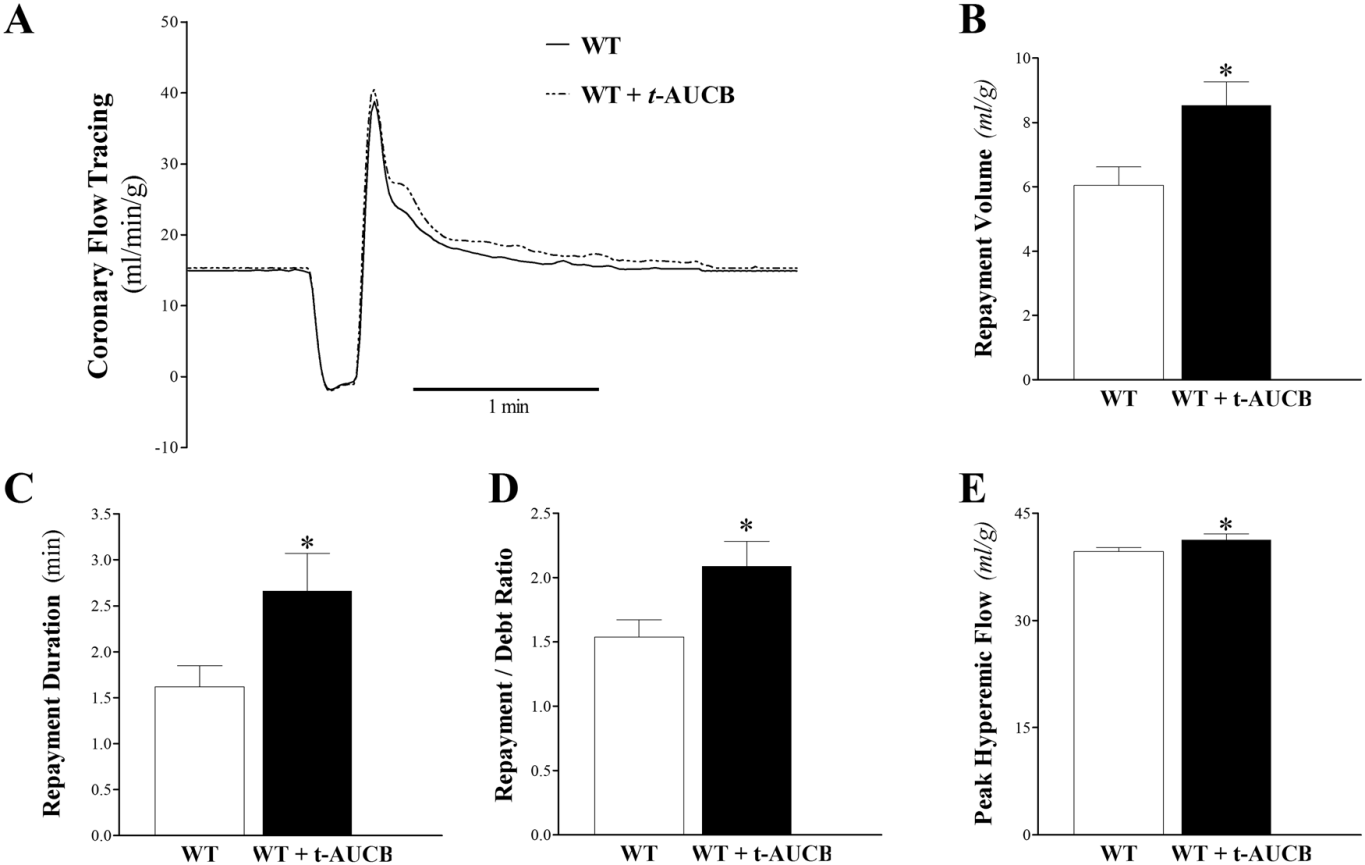
Comparison of coronary reactive hyperemia (CRH) before (WT) and after (*t-*AUCB-treated WT) infusion of *t*-AUCB. (A) tracing depicting coronary flow (CF) changes at baseline (before CRH) and after CRH was induced by 15-second no-flow ischemia in WT (continuous line) and WT + *t*-AUCB (dashed line). Repayment volume (B), repayment duration (C), repayment/debt ratio (D), and peak hyperemic flow (E) increased in *t-*AUCB-treated WT versus WT mice (p < 0.05). * p < 0.05 versus WT. *n* = 8.

*t*-AUCB enhanced CRH in WT mice (Fig 1A). Compared to WT mice, *t-*AUCB-treated WT mice had increased RV (41%; 6.1 ± 0.5 and 8.5 ± 0.4 mL/g, respectively; p < 0.05, Fig 1B), increased RD (64%; 1.6 ± 0.2 and 2.7 ± 0.4, respectively; Fig 1C), an increased repayment/debt (R/D) ratio (36%; 1.5 ± 0.1 and 2.1 ± 0.2, respectively; p < 0.05; Fig 1D), and slightly increased PHF (39.7 ± 0.7 and 41.2 ± 1.0 mL/min/g respectively, p < 0.05; Fig 1E). Baseline CF, LVPD, and HR were not different between the two groups (p > 0.05). Time-matched control experiments with WT mouse hearts, employing three consecutive inductions of CRH, showed no change in the CRH response and no difference in baseline heart functions, including CF, LVDP, and HR (data not shown).

#### Effect of *t*-AUCB on CRH Response in sEH^–/–^Mice

**Fig 2:**
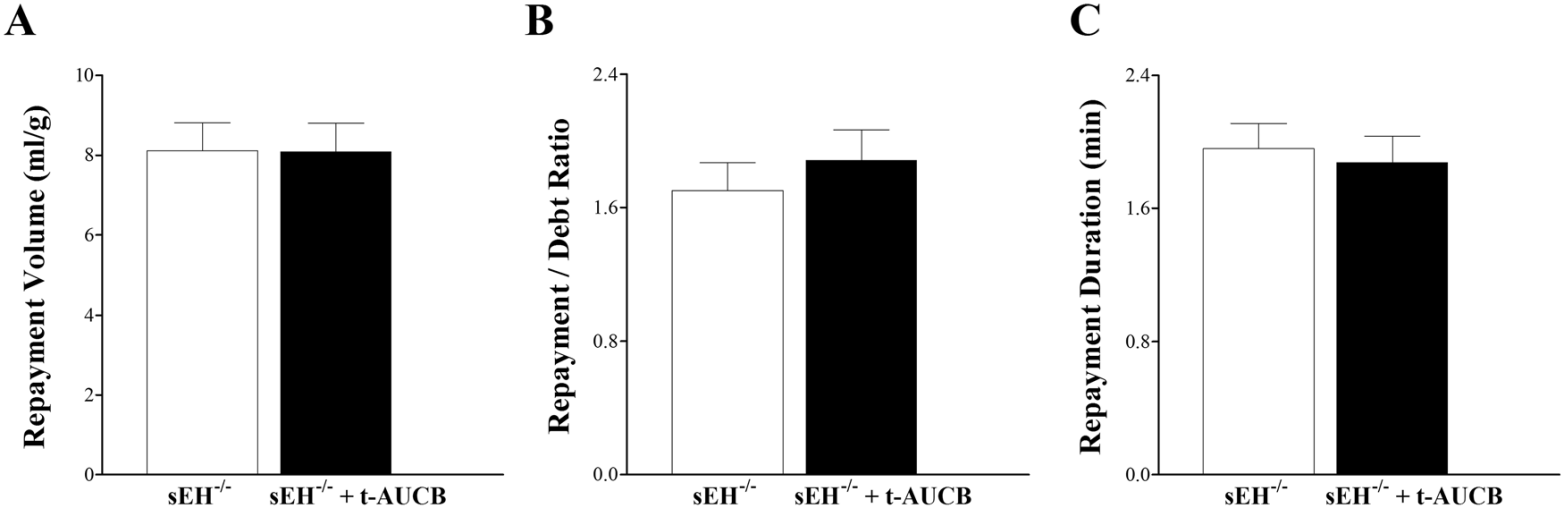
Effect of sEH-inhibitor, *t*-AUCB, on coronary reactive hyperemia (CRH) in sEH-null (sEH^–/–^) mice. *t*-AUCB did not (p > 0.05) affect CRH in sEH^–/–^mice, as evident in the unchanged repayment volume (A), repayment/debt ratio (B), and repayment duration (C) (p = 0.690). *n* = 6.

Using the same experimental protocol as in the preceding section, *t*-AUCB did not have a significant effect on CRH in sEH^–/–^mice, including RV (p > 0.05, Fig 2A), R/D (p > 0.05, Fig 2B), RD (p > 0.05, Fig 2C), baseline CF, PHF, HR, or LVDP (data not shown).

### Oxylipin Analysis of Heart Perfusate before and after *t*-AUCB infusion in WT Mice

**Fig 3:**
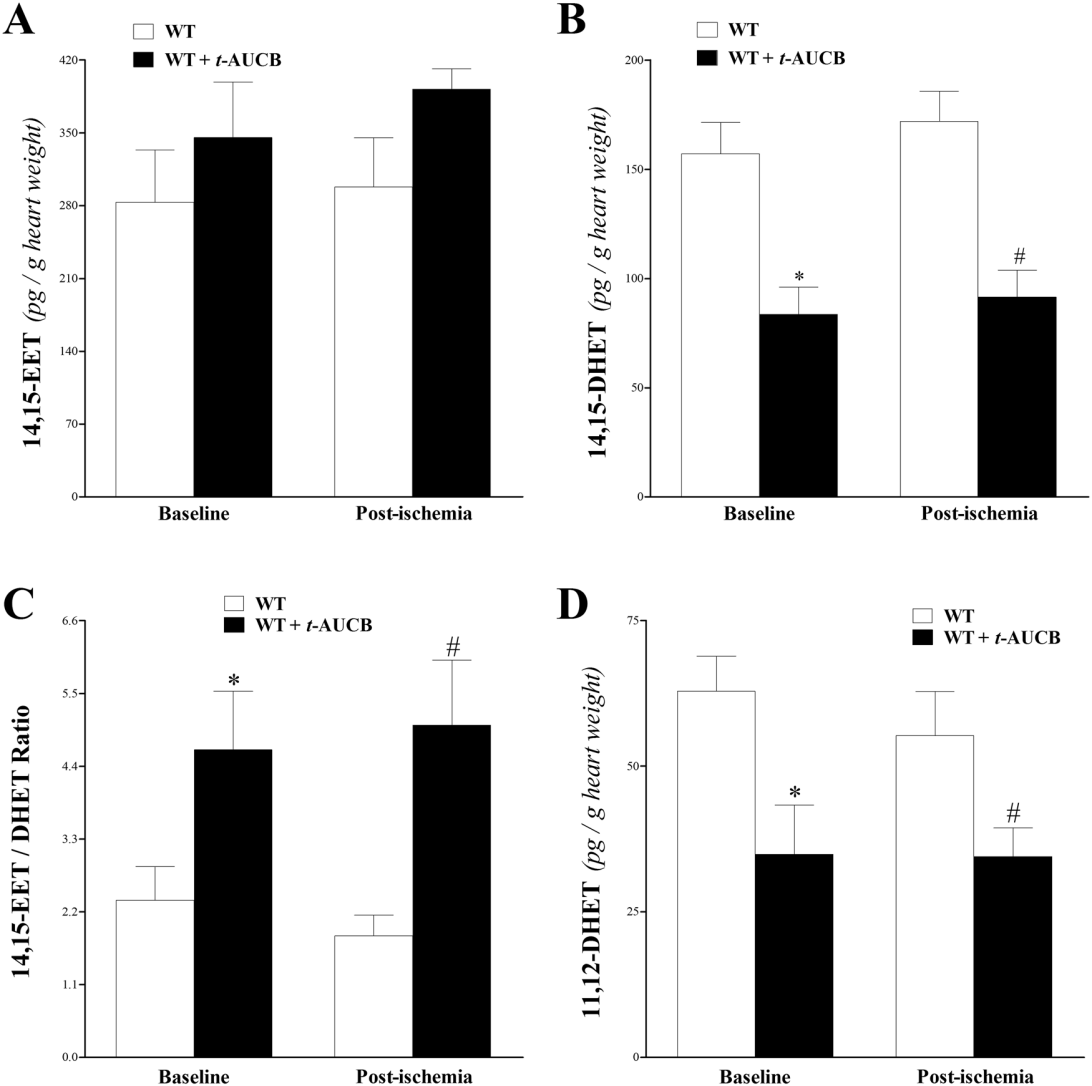
LC–MS/MS analysis for 14, 15–EET, 14, 15–DHET, and 11,12–DHET levels in WT and *t-*AUCB-treated WT mouse heart perfusates at baseline (pre-ischemia) and directly after 15-second ischemia (post-ischemia). (A) At baseline and post-ischemia levels of 14, 15–EET had an increasing trend in *t-*AUCB-treated WT versus WT mice, but this trend was not significant. (B) 14, 15–DHET levels decreased at baseline and post-ischemia in *t-*AUCB-treated WT versus WT (p < 0.0001). (C) The 14, 15–EET/14, 15–DHET ratio increased (p < 0.05) in *t-*AUCB-treated WT versus WT mice at baseline (by 96%) and post-ischemia (by 173%). (D) 11, 12–DHET levels decreased (p < 0.001) at baseline and post-ischemia in *t-*AUCB-treated-WT versus WT mice. There was no difference in 14,15-EET, 14,15-DHET, and 11,12-DHET levels pre- and post-ischemia within each group. * p < 0.05 versus baseline WT. # p < 0.05 versus post-ischemia WT. *n* = 8.

Heart perfusate oxylipin levels were determined by LC–MS/MS. Perfusate samples were collected at baseline after stabilization and right after ischemia in WT and *t*-AUCB-treated WT mice. Out of the four EET regioisomers, only 14,15-EET, its corresponding metabolite (14,15-DHET), and 11,12-DHET were detected. An increasing trend in the level of 14,15-EET in *t-*AUCB-treated WT versus WT mice was observed at baseline and post-ischemia, but was not significant (p > 0.05, Fig 3A). However, sEH-metabolized 14,15-DHET significantly decreased in *t-*AUCB-treated WT versus WT mice at baseline and post-ischemia (p < 0.0001, Fig 3B). As a result, the ratio of 14,15-EET/DHET increased in *t-*AUCB-treated WT versus WT mice at baseline (by 96%) and post-ischemia (by 173%; p < 0.05, Fig 3C). Our technique also detected 11,12-DHET, which decreased in *t-*AUCB-treated WT versus WT mice at baseline and post-ischemia (p < 0.001, Fig 3D). There was no differences in levels of 14,15-EET, 14,15-DHET, or 11,12-DHET pre- and post-ischemia within each group.

**Fig 4:**
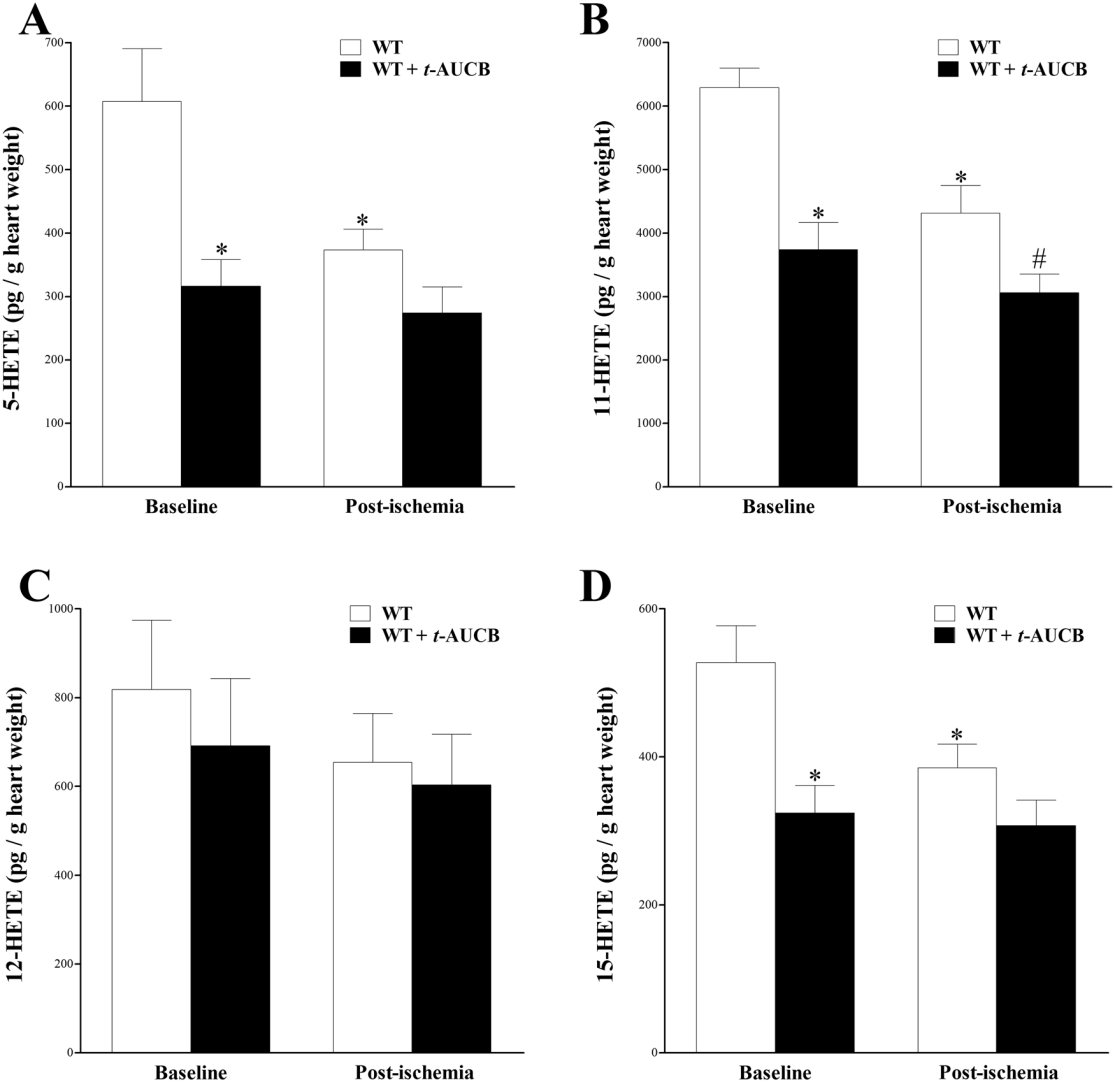
LC–MS/MS analysis of 5-, 11-, 12- and 15-HETE levels in WT and *t-*AUCB-treated WT mouse heart perfusates at baseline (pre-ischemia) and post-ischemia. In WT mice, 5-, 11-, 12- and 15-HETE levels decreased post-ischemia compared to baseline, but this was only significant for 5-HETE (A), 11-HETE (B), and 15-HETE (D) (p < 0.05). The same mid-chain HETEs had a decreasing trend post-ischemia compared to baseline in *t-*AUCB-treated WT mice, but this was not significant (p > 0.05, A-D). Treatment with *t*-AUCB decreased HETE levels in WT mice, which was significant for 5-HETE (A), 11-HETE (B) and 15-HETE (D) levels at baseline (p < 0.05), and in 11-HETE levels post-ischemia (p < 0.05, B). * p < 0.05 versus baseline WT. # p < 0.05 versus post-ischemia WT. *n* = 8.

Our LC–MS/MS detected 4 mid-chain HETEs (5-, 11-, 12-, and 15-HETE) in WT and *t-*AUCB-treated WT mouse heart perfusates. In WT mice, levels of 5-, 11-, 12-, and 15-HETE decreased post-ischemia (after perfusion was reinstated) compared to baseline, but this was only significant for 5-, 11-, and 15-HETE (p < 0.05, Fig 4A, 4B and 4D). These mid-chain HETEs had a decreasing trend post-ischemia compared to baseline in *t-*AUCB-treated WT mice, but this trend was not significant (p > 0.05, Fig 4A–4D). Treatment with *t*-AUCB decreased HETE levels in WT mice, which was significant for 5-, 11-, and 15-HETE at baseline (p < 0.05, Fig 4A, 4B and 4D) and 11-HETE post-ischemia (p < 0.05, Fig 4B).

**Fig 5:**
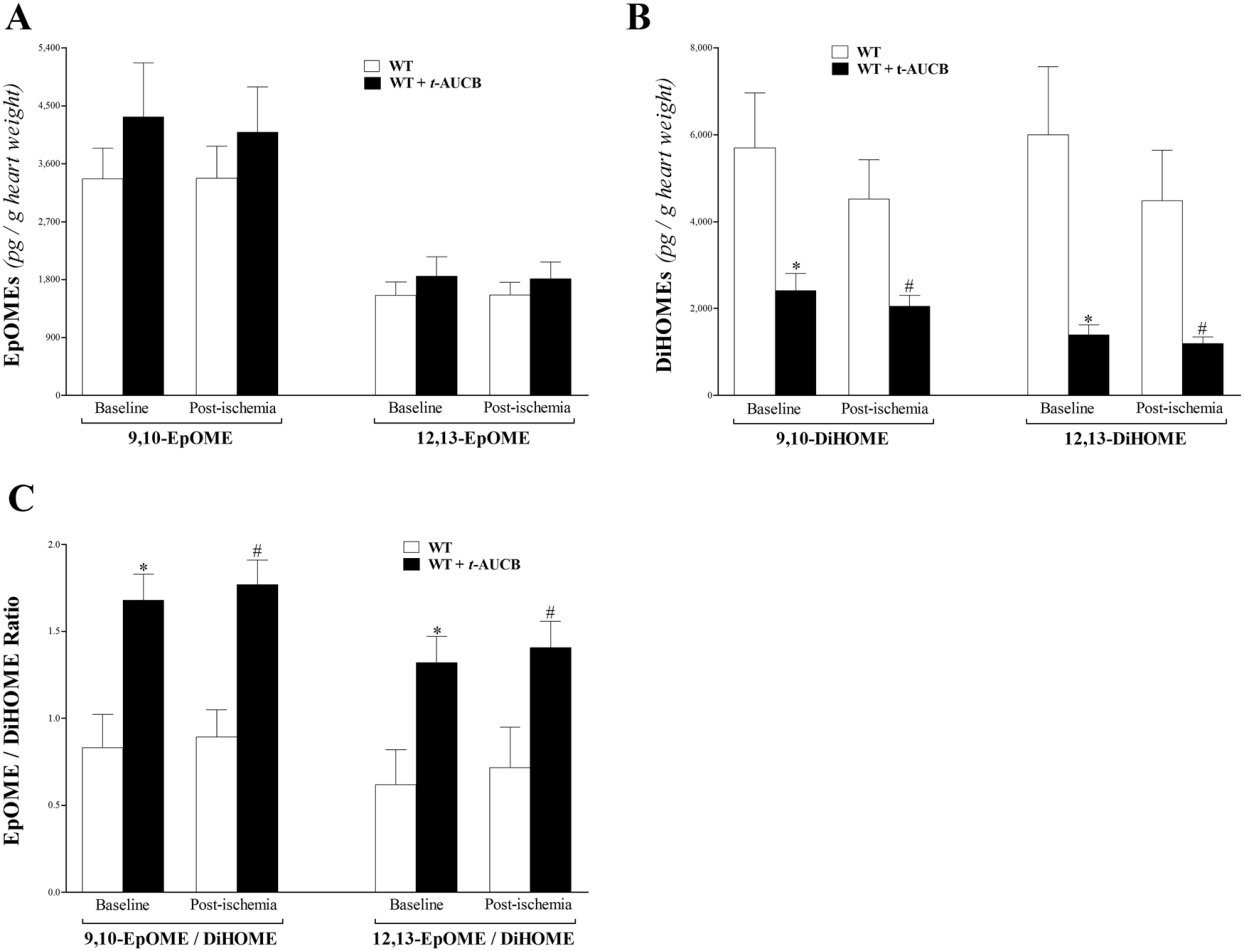
LC–MS/MS analysis of EpOME and DiHOME levels and the EpOME/DiHOME ratio in WT and *t-*AUCB-treated-WT mouse heart perfusates at baseline (pre-ischemia) and post-ischemia. (A) 9,10- and 12,13-EpOME levels had an increasing trend at baseline and post-ischemia in *t-*AUCB-treated-WT versus WT mice, but this was not significant (p > 0.05). Neither EpOME was significantly changed post-ischemia compared to baseline in both groups. (B) 9,10- and 12,13-DiHOME levels decreased at baseline and post-ischemia in *t-*AUCB-treated-WT versus WT mice (p < 0.001). (C) The EpOME/DiHOME ratio increased in *t-*AUCB-treated versus WT mice at baseline and post-ischemia (p < 0.0001). The measured EpOME and DiHOME levels and EpOME/DiHOME ratio did not change post ischemia versus baseline within the same group (p > 0.05, A-C). * p < 0.05 versus baseline WT. # p < 0.05 versus post-ischemia WT. *n* = 8.

Linoleic acid (LA) epoxides (9,10- and 12,13-EpOME) levels had an increasing trend at baseline and post-ischemia in *t-*AUCB-treated WT versus WT mice, but was not significant (p > 0.05, Fig 5A). The corresponding 9, 10- and 12, 13-DiHOME levels decreased at baseline and post-ischemia in *t-*AUCB-treated WT versus WT mice (p < 0.001, Fig 5B). As a result, the EpOME/DiHOME ratio increased in *t-*AUCB-treated WT compared to WT mice at baseline and post-ischemia (p < 0.0001, Fig 5C). The measured EpOMEs, DiHOMEs, and EpOME/DiHOME ratio did not change post-ischemia versus baseline within the same group (p > 0.05, Fig 5A–5C).

**Fig 6:**
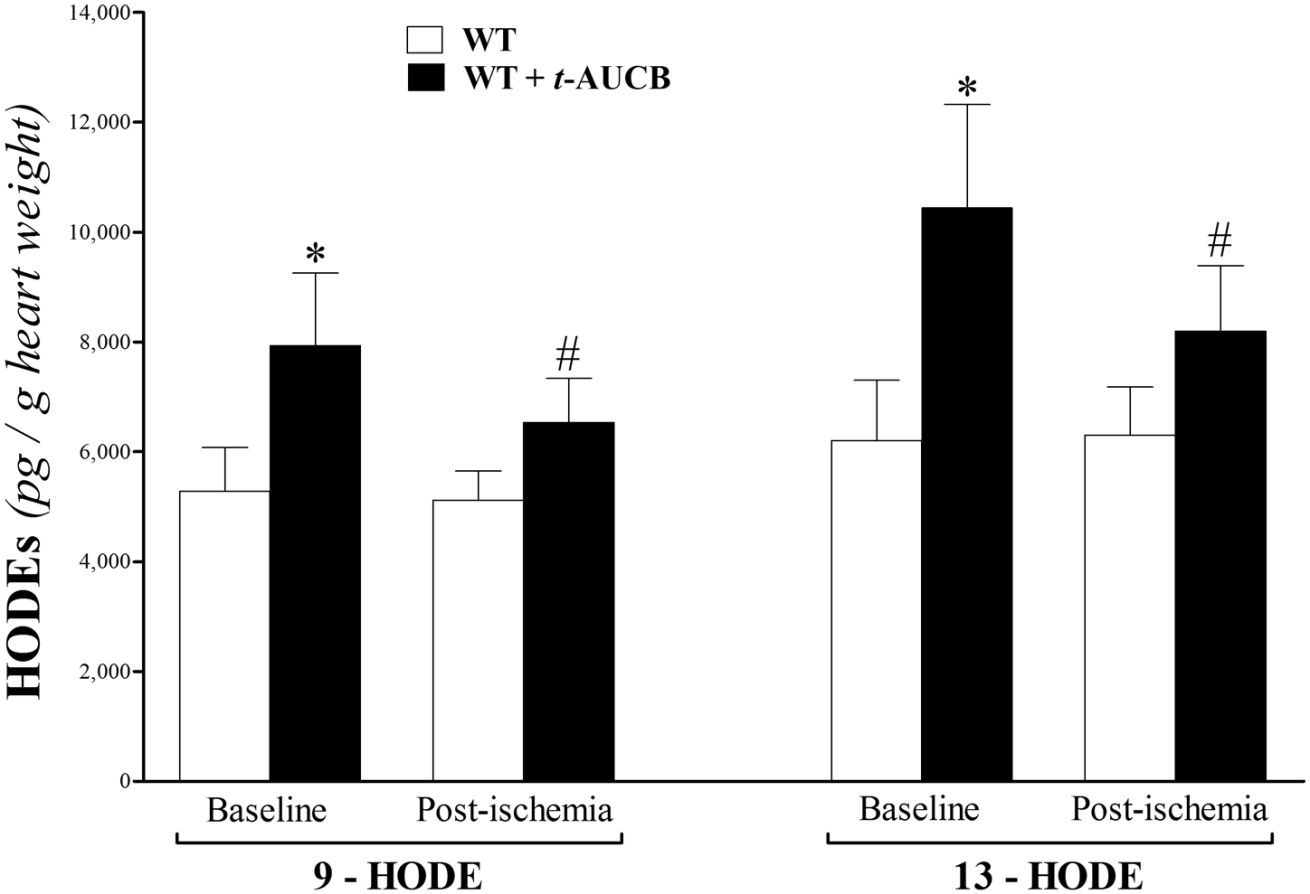
LC–MS/MS analysis of HODEs in WT and *t-*AUCB-treated WT mouse heart perfusates at baseline (pre-ischemia) and post-ischemia. 9- and 13-HODE increased in *t-*AUCB-treated WT versus WT mice at baseline and post-ischemia (p < 0.05). Neither HODE changed post-ischemia versus baseline within the same group (p > 0.05). * p < 0.05 versus baseline WT. # p < 0.05 versus post-ischemia WT. *n* = 8.

Other LA hydroxylated metabolites, 9- and 13-HODE, increased in *t-*AUCB-treated WT versus WT mice at baseline and post-ischemia (p < 0.05, Fig 6); however, neither HODE level changed post-ischemia versus baseline within the same group (p > 0.05, Fig 6).

**Fig 7:**
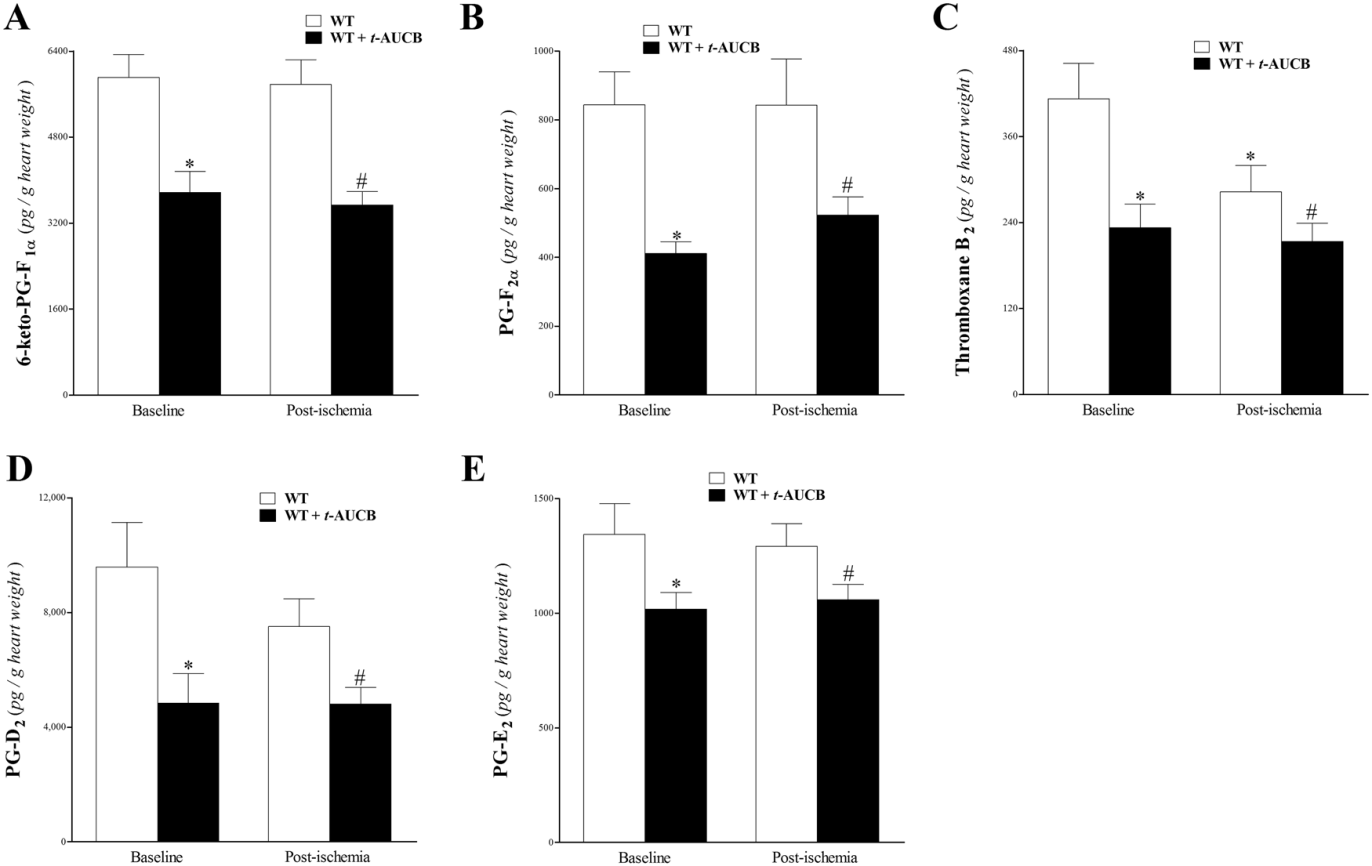
LC–MS/MS analysis of 6-keto-PG-F_1α_, PG-F_2α_, thromboxane B_2_, PG-D_2_, and PG-E_2_ in WT and *t-*AUCB-treated WT mouse heart perfusates at baseline (pre-ischemia) and post-ischemia. Infusion of *t*-AUCB decreased 6-keto-PG-F_1α_ (A), PG-F_2α_, (B), thromboxane B_2_ (C), PG-D_2_ (D), and PG-E_2_ (E), at baseline and post-ischemia (p < 0.05). Compared to baseline WT, post-ischemia WT levels were decreased for thromboxane B_2_, and PG-D_2_, but were significant (p < 0.05) only for thromboxane B_2_ (C). * p < 0.05 versus baseline WT. # p < 0.05 versus post-ischemia WT. *n* = 8.

The levels of 6K-PG-F_1α_, PG-F_2α_, TxB_2_, PG-D_2_, and PG-E_2_ was also detected by our LC–MS/MS. Treatment of WT mouse hearts with *t*-AUCB at baseline and post-ischemia decreased 6-keto-PG-F_1α_ (p < 0.05, Fig 7A), PG-F_2α_ (p < 0.05, Fig 7B), TxB_2_ (p < 0.05, Fig 7C), PG-D_2_ (p < 0.05, Fig 7D), and PG-E_2_ (p < 0.05, Fig 7E). Compared to baseline WT levels, post-ischemia WT levels decreased for TxB_2_ and PG-D_2_, but were significant (p < 0.05) only for TxB_2_ (Fig 7C).

### Effect of MS-PPOH (CYP epoxygenase inhibitor) on CRH Response in WT Mice

**Fig 8:**
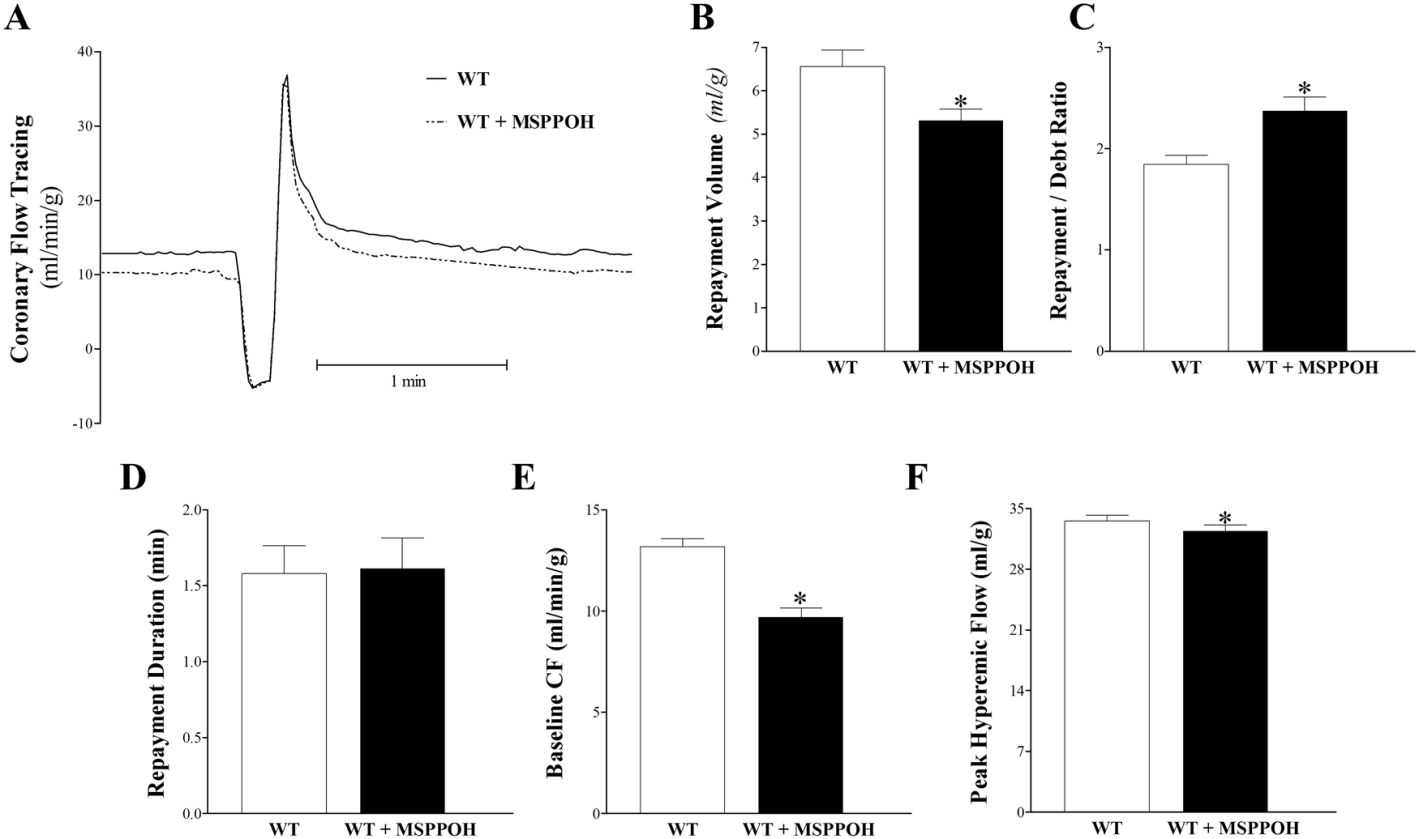
Effect of CYP-epoxygenase inhibitor (MS-PPOH, 1 μM) on coronary reactive hyperemia (CRH) in wild type (WT) mice. Each WT isolated mouse heart was used as its own control. (A) tracing depicting coronary flow changes at baseline (before CRH) and after CRH was induced by 15-second no-flow ischemia in WT (continuous line) and MS-PPOH-treated WT (dashed line) mice. Repayment volume (B), baseline CF (E), PHF (F), and LVDP (G) decreased (p < 0.05) in MS-PPOH-treated WT versus WT mice. Repayment/debt ratio (C) increased (p < 0.05), whereas repayment duration (D) did not change (p > 0.05) after in MS-PPOH-treated WT versus WT mice. * p < 0.05 versus WT. *n* = 8.

MS-PPOH attenuated CRH in WT mice (Fig 8A). RV was decreased by 19% in MS-PPOH-treated WT versus WT mice (6.6 ± 0.4 and 5.3 ± 0.4 mL/g respectively, p < 0.05, Fig 8B). Baseline CF was also decreased by 26% in MS-PPOH-treated WT versus WT mice (13.2 ± 0.4 and 9.7 ± 0.6 mL/g respectively, p < 0.05, Fig 8E). As a result, the debt volume (calculated as the area under the CF curve during the 15 second ischemia) was decreased and, subsequently, the R/D ratio was increased in MS-PPOH-treated WT compared to WT mice (1.9 ± 0.1 and 2.4 ± 0.2 respectively, p < 0.05, Fig 8C). PHF was slightly decreased in MS-PPOH-treated WT versus WT mice (33.6 ± 0.7 and 32.4 ± 0.7 mL/min/g respectively, p < 0.05, Fig 1F). RD (Fig 8D), HR, and LVPD were not different between the two groups (p > 0.05).

### Effect of T0070907 (PPARγ antagonist) on CRH response in WT Mice

**Fig 9:**
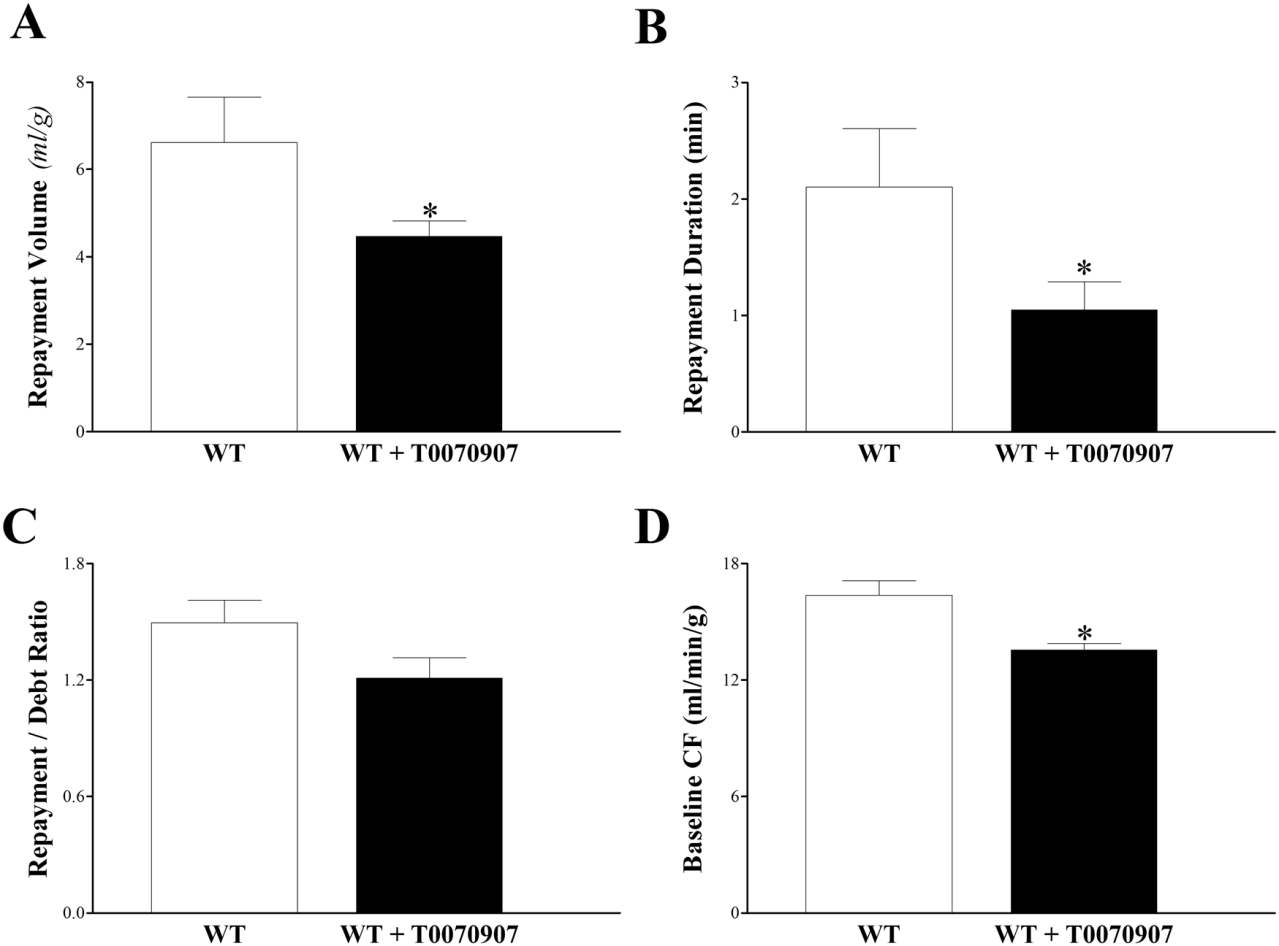
Effect of T0070907, PPARγ-antagonist (10 μM) on coronary reactive hyperemia (CRH) in wild type (WT) mice. Each WT isolated mouse heart was used as its own control. Infusion of T0070907 into WT mice hearts attenuated CRH. (A) Repayment volume decreased (p < 0.05), as did RD (B) (p < 0.05) and baseline CF (D) (p < 0.05). Repayment/debt ratio (C) had a decreasing trend, but was not significant (p > 0.05). * p < 0.05 versus WT. *n* = 8.

Administering T0070907 to WT mouse hearts attenuated CRH. In T0070907-treated WT versus WT mice, RV was decreased by 32% (from 6.6 ± 0.7 to 4.5 ± 0.6 mL/g, p < 0.05, Fig 9A), RD by 50% (from 2.1 ± 0.5 to 1.1 ± 0.2 min, p < 0.05, Fig 9B), and baseline CF by 10% (from 16.3 ± 0.7 to 15.1 ± 1.5 mL/min/g, p < 0.05, Fig 9D). The R/D ratio decreased from 1.7 ± 0.3 to 1.2 ± 0.1, but was not significant (p > 0.05, Fig 9C). LVDP, PHF, and HR were not different between the two groups (p > 0.05, data not shown).

### Effect of T0070907on *t*-AUCB–enhanced CRH in WT Mice

**Fig 10:**
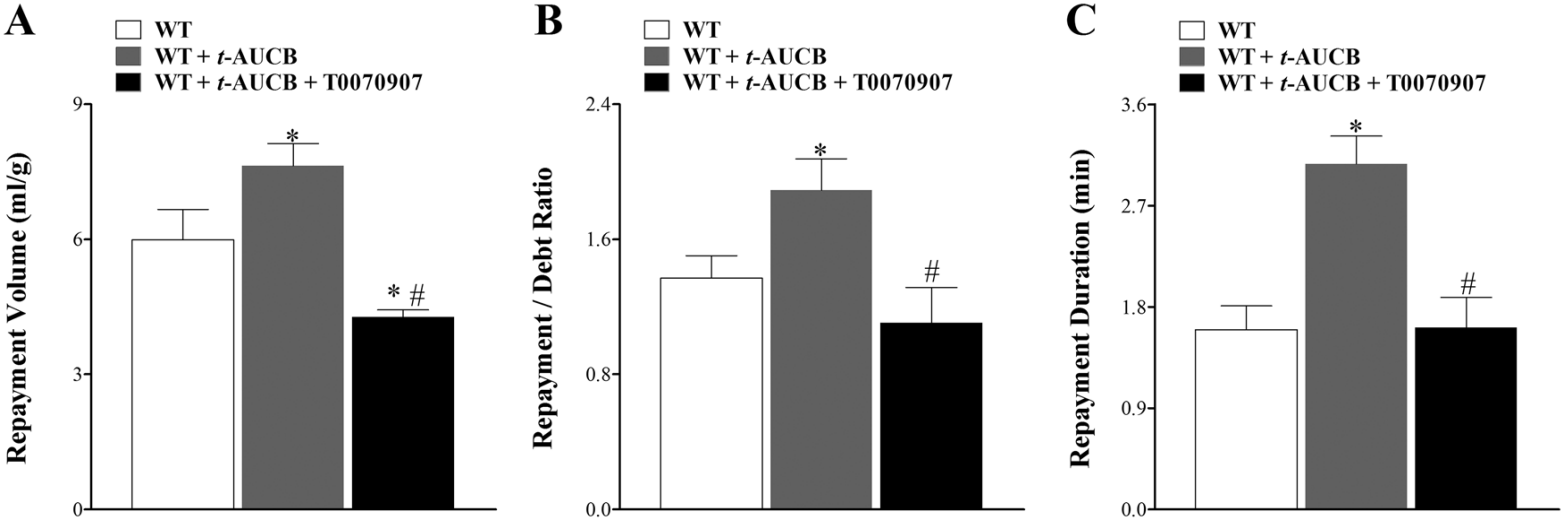
Effect of T0070907, PPARγ-antagonist (10 μM) on *t*-AUCB-enhanced CRH in WT in wild type (WT) mice. Each WT isolated mouse heart was used as its own control. Inhibition of sEH by *t*-AUCB enhanced CRH in WT mice. Infusion of *t*-AUCB (10 μM) increased repayment volume (RV) (p < 0.05, A), repayment/debt ratio (p < 0.05, B), and repayment duration (RD) in WT mice (p < 0.05, C). The *t*-AUCB-enhanced CRH was attenuated by T0070907. T0070907 decreased RV (A), R/D ratio (B), and RD (C) in *t-AUCB-treated-WT mice* (p < 0.05). * p < 0.05 versus WT. *n* = 6.

Pharmacologic inhibition of sEH by *t*-AUCB enhanced CRH in WT mice, as mentioned earlier (Fig 1). This same experiment was repeated with the PPARγ-antagonist T0070907 added to study its effect on *t*-AUCB-enhanced CRH. Infusion of *t*-AUCB increased RV in WT mice (from 6.0 ± 0.7 to 7.6 ± 0.5 mL/g, p < 0.05, Fig 10A), R/D ratio (from 1.4 ± 0.1 to 1.9 ± 0.2, p < 0.05, Fig 10B), and RD (from 1.6 ± 0.2 to 3.1 ± 0.3, p < 0.05, Fig 10C). *t*-AUCB–enhanced CRH was attenuated by T0070907. Addition of T0090709 to *t-*AUCB-treated WT mice decreased RV (from 7.6 ± 0.5 to 4.3 ± 0.2 mL/g, p < 0.05, Fig 10A), R/D ratio (from 1.9 ± 0.2 to 1.1 ± 0.2, p < 0.05, Fig 10B) and RD (from 3.1 ± 0.3 to 1.6 ± 0.3, p > 0.05, Fig 10C). Baseline CF, PHF, HR and LVDP were not significantly changed among the 3 groups (WT, [WT + *t*-AUCB], and [WT + *t*-AUCB + T0070907], data not shown).

### Effect of rosiglitazone (PPARγ agonist) on CRH Response in WT Mice

**Fig 11:**
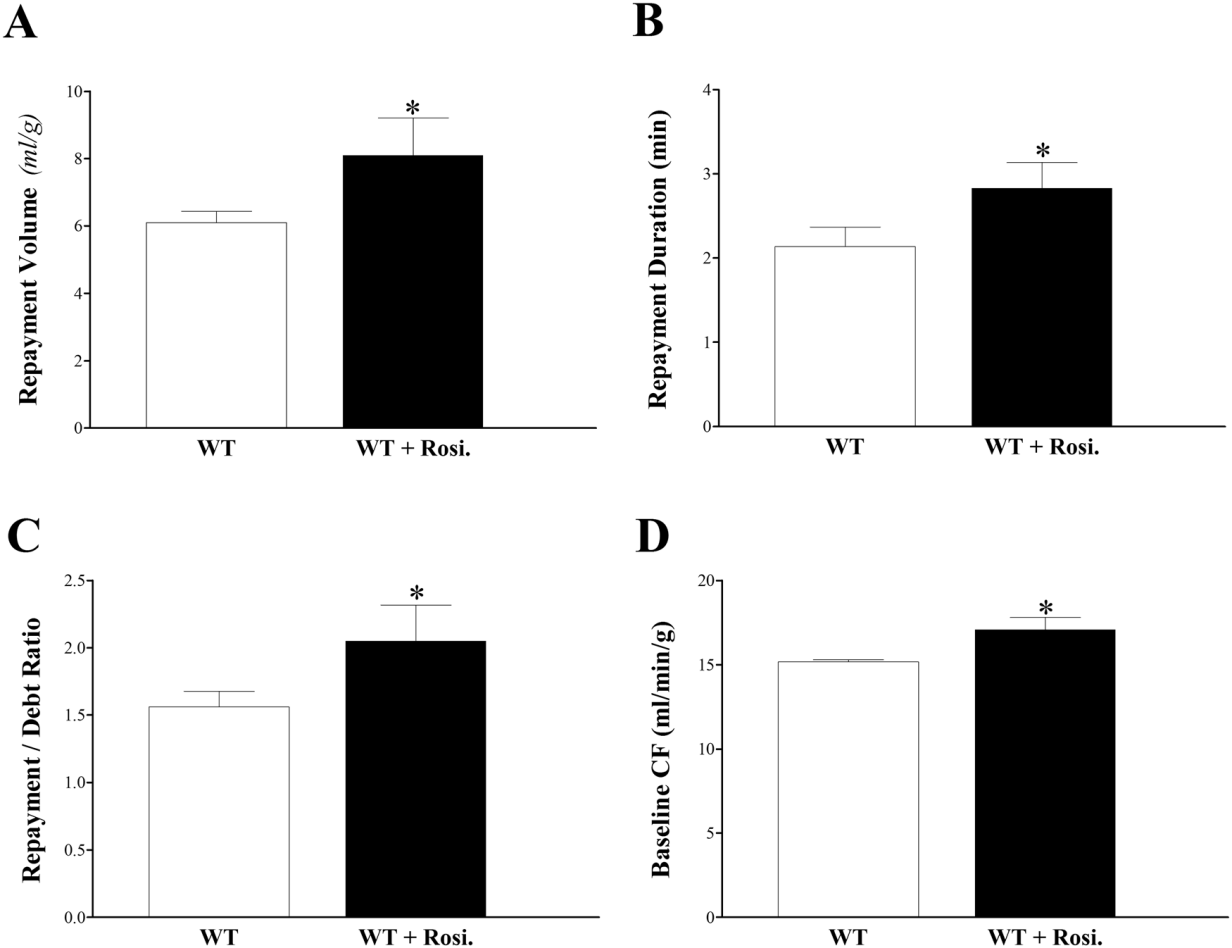
Comparison of CRH in WT before and after infusion of rosiglitazone (PPARγ-agonist, 10 μM). Each isolated heart was used as its own control. Repayment volume (A), repayment duration (B), repayment/debt ratio (C), and baseline CF (D) increased after rosiglitazone administration (p < 0.05). * p < 0.05 versus WT. *n* = 8.

**Fig 12:**
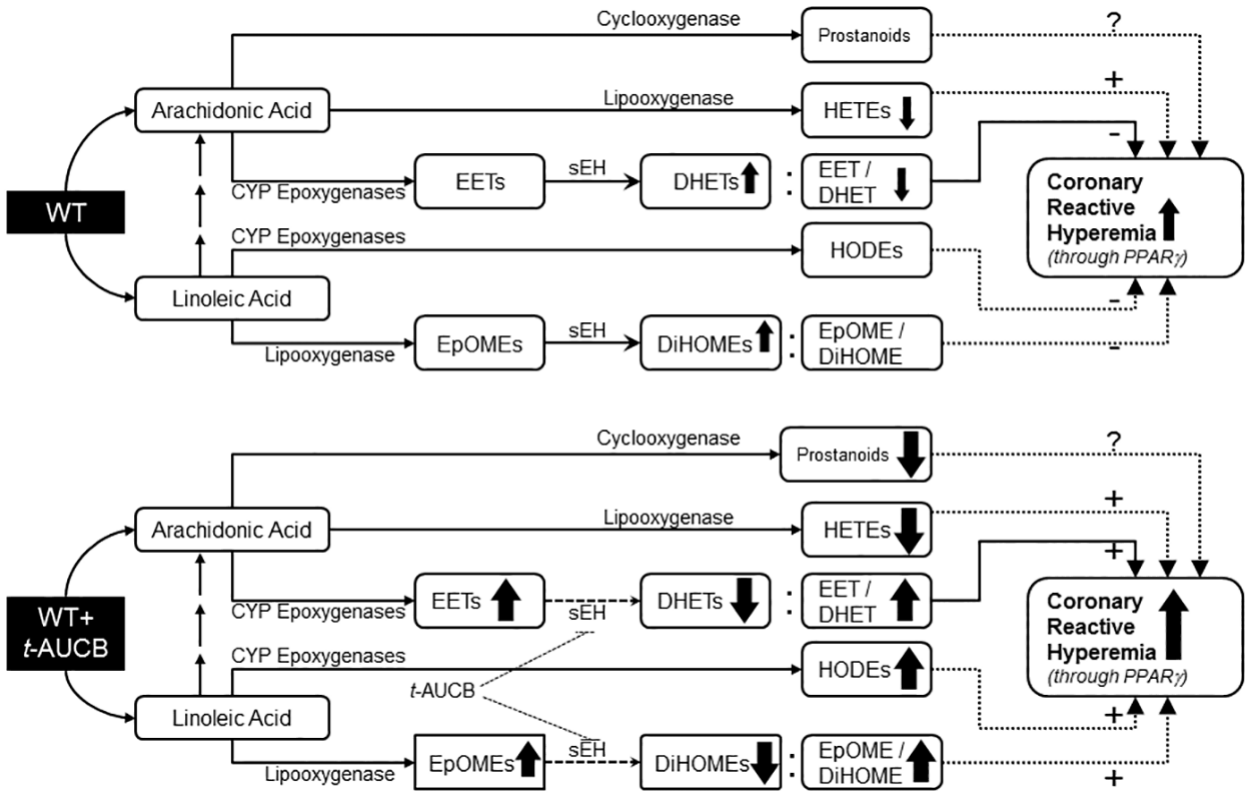
A schematic diagram comparing the oxylipin changes observed in response to a brief ischemia and their possible impact on coronary reactive hyperemia (CRH) between WT and *t*-AUCB-treated WT mice. Treatment with *t*-AUCB enhanced CRH possibly through increased EET/DHET ratio, increased 13-HODE, increased EpOME/DiHOME ratio, decreased mid-chain HETEs, and PPARγ activation.

Infusion of rosiglitazone enhanced CRH in WT mice. In rosiglitazone-treated WT versus WT mice, RV increased by 33% (from 6.1 ± 0.3 to 8.1 ± 0.9 mL/g, p < 0.05, Fig 11A), RD by 33% (from 1.6 ± 0.1 to 2.1 ± 0.3 min, p < 0.05, Fig 11B), R/D ratio by 31% (from 1.6 ± 0.1 to 1.9 ± 0.2, p < 0.05, Fig 11C), and baseline CF from 15.2 ± 0.1 to 17.1 ± 0.7 mL/min/g (p < 0.05, Fig 11D). PHF, LVDP and HR were not different between the two groups (p > 0.05, data not shown). We summarized our observed results into a proposed schematic diagram (Fig 12).

## Discussion

Pharmacologic inhibition of sEH in WT mice enhanced CRH in an isolated heart model after brief ischemia. sEH inhibition was also associated with changes in various oxylipin profiles. The relationship among inhibition of CYP epoxygenases, inhibition of sEH, oxylipins, and PPARγ in the modulation of CRH in isolated mouse hearts is not known. Therefore, this study was designed to investigate the role of sEH, oxylipins, and PPARγ in the modulation of CRH using isolated WT mouse hearts. Our data demonstrated that: **1)** Inhibition of sEH by *t*-AUCB enhanced CRH; **2)** Treatment with *t*-AUCB significantly changed oxylipin profiles, including an increased EET/DHET ratio, increased EpOME/DiHOME ratio, increased HODEs, decreased mid-chain HETEs, and decreased prostanoids; **3)** Inhibition of CYP epoxygenases (by MS-PPOH) attenuated CRH; **4)** The PPARγ antagonist T0070907 decreased CRH, and **5)** the PPARγ agonist rosiglitazone increased CRH.

Inhibition of sEH by *t*-AUCB significantly enhanced CRH after brief ischemia compared to non-treated WT mice. The association between compromised CRH and some cardiovascular pathologies [26–28] confirms the significance of CRH. Further, the *t*-AUCB-enhanced CRH was accompanied by a 1.0- (at baseline) to 1.7- (post-ischemia) fold increase in the 14,15-EET/DHET ratio, primarily driven by a decrease in 14,15-DHET. Although, a trend of increase in 14,15-EET level was observed in *t*-AUCB–treated vs. non-treated WT mice, the difference was not statistically significant. This could be attributed to several factors including the acute use of *t*-AUCB for a short period, the short half-life of EETs compared to DHETs, and the short duration (15 seconds) of ischemic insult. Another dihydroxy-EET metabolite (11,12-DHET) decreased in *t*-AUCB–treated mouse hearts, further confirming the efficacy of the sEH-inhibitor, *t*-AUCB. The specificity of *t*-AUCB as a sEH inhibitor was tested using sEH knockout (sEH^–/–^) mice, where no difference in CRH between the *t*-AUCB-treated sEH^–/–^versus non-treated sEH^–/–^mice was observed. The rapid hydration of EETs [37] may explain the challenge of detecting them. We were able to detect 14,15-EET and two diols (11,12- and 14,15-DHETs) in heart perfusate samples. As mentioned earlier, EETs are formed by the epoxidation of arachidonic acid (AA) by CYP epoxygenases and possess well-established beneficial cardiovascular effects [7, 38–41], including protection against ischemia/reperfusion injury [5], and vasodilation in many vascular beds such as the intestines [42], kidney preglomerular vasculature [43], and brain [44]. Inhibition of sEH, the main catabolic enzyme of EETs, has been widely used to increase the level of EETs and to investigate their effects [11]. Acute inhibition of sEH, through injection of a sEH inhibitor, lowered blood pressure in spontaneously hypertensive rats (SHR) [45], whereas chronic inhibition of sEH lowered angiotensin-II–induced hypertension [46]. Besides lowering blood pressure, sEH inhibition ameliorated renal damage in angiotensin-dependent, salt-sensitive hypertension rats [47]. In the current study, the brief ischemia had no effect on EET or DHET levels. This could be due to the short duration of ischemia (15 seconds). In contrast, DHETs (EETs metabolites) were elevated in the mouse heart perfusate in response to longer (20-min) ischemia [6]. Another interesting finding in the current study was that no significant difference was found in CRH between male and female mice in either *t*-AUCB–treated or non–treated groups (data not presented). Based on our data, the enhancement of CRH associated with sEH inhibition in WT mice may be partially due to an increase in the EET/DHET ratio.

Mid-chain (5-, 11-, 12- and 15-) HETE levels decreased in response to ischemia and to *t*-AUCB treatment at baseline and post-ischemia. Mid-chain HETEs are produced through allylic oxidation of AA by lipoxygenase (LOX) [48]. They were shown to have chemotaxis effects, change vascular tone, and induce the production of vascular endothelial growth factors [16–19]. Also, the increased formation of mid-chain HETEs was involved in cardiovascular dysfunction [49–52]. Unlike the vasodilatory effect of EETs in the kidneys [43], 12-HETE caused vasoconstriction in small renal arteries [53]. In human umbilical artery smooth muscle cells, 11-HETE inhibited vascular smooth muscle cell proliferation [54], whereas in the isolated Guinea Pig lungs, 5- and 15-HETEs produced vasoconstriction and edema [55]. Also, the generation of mid-chain HETEs is increased in essential hypertension [56] suggesting that they could be involved in its pathogenesis. These reports point to opposite effects of EETs and HETEs in vascular biology. Maayah et al. reported that mid-chain HETEs blocked the synthesis of EETs and increased their conversion to DHETs in RL-14 cells [57]. Moreover, although sEH is not directly involved in the generation or breakdown of mid-chain HETEs, sEH was found to be essential for mid-chain HETE–mediated induction of cellular hypertrophy [57]. Therefore, not only do EETs and mid-chain HETEs have opposite effects, they seem to affect the level of each other. In contrast to our findings, Li et al. reported that the plasma levels of 5-, 11-, 12-, and 15-HETE were not changed by sEH-inhibition in WT mice [11]. However, their results were based on plasma samples, whereas ours were based on isolated heart perfusate samples and demonstrated significant decrease in the level of the same HETEs in response to sEH inhibition. Therefore, in this study, it is possible that the decrease in mid-chain HETEs induced by sEHi (*t*-AUCB) has also contributed to blocking the enhanced conversion of EETs to DHETS by HETEs. This in turn would contribute to the enhanced CRH through an increase in EET/DHET ratio. Based on these results, the decrease in mid-chain HETEs in both *t*-AUCB-treated and non-treated mice in response to ischemia suggests that these metabolites are down regulated in response to ischemic episodes. This is an interesting finding because HETEs were the only oxylipins analyzed in this paper whose levels were affected by the brief ischemia. Also, the further decrease of these HETEs by *t*-AUCB–treatment may have played a role in the enhanced CRH observed in *t*-AUCB–treated hearts.

Linoleic acid (LA) epoxidation metabolites, such as 9,10-, and 12,13-EpOMEs, and their corresponding sEH-metabolized DiHOMEs were detected in the heart perfusate samples. As expected, inhibition of sEH by *t*-AUCB was further confirmed by an increased EpOME/DiHOME ratio. This resulted from increased 9,10-,12,13-EpOMEs (albeit not statistically significant), and decreased 9,10-, 12,13-DiHOMEs in *t*-AUCB–treated versus non-treated mice. The physiological significance of EpOMEs and DiHOMEs remains poorly understood, and the evidence is somewhat contradictory, with a few studies suggesting toxic effects, while others indicating beneficial effects of these bioactive metabolites. EpOMEs and DiHOMEs were reported to increase oxidative stress in vascular endothelial cells [58]; DiHOMEs were toxic to renal proximal tubular cells [59]; and intravenously injected 9,10-EpOME had cardiodepressive effects in dogs [60]. The main caveat of these studies, which reported that EpOMEs and DiHOMEs have toxic effects, is that very high concentrations (100–500 μM) were used [48]. Contrary to these reports, lower, more physiological concentrations of EpOMEs and DiHOMEs did not have toxic effects and rather had beneficial effects [61]. Mitchell et al reported that LA and its oxidative metabolites (EpOMEs and DiHOMEs) did not have toxic effects during acute exposure in Langendorff-perfused rat hearts [61]. Pretreatment with 12,13-EpOME protected primary cultures of rabbit renal proximal tubular cells against hypoxia/reoxygenation injury [62]. Also, the increased EpOME/DiHOME ratio, as induced by sEH inhibition using AUDA, improved renal recovery in response to ischemia/reperfusion injury in C57BL/6 mice [14]. Based on these findings and on the described biologic role of LA epoxides, the increase in EpOME/DiHOME ratio in *t*-AUCB–treated mouse heart perfusates may have contributed to enhancing CRH.

Linoleic acid (LA) is also metabolized through hydroxylation by CYP epoxygenases to form hydroxyl-LA metabolites known as hydroxyoctadecadienoic acids (HODEs) [48]. The detected two HODE isomers, 9-, and 13-HODE, were increased in *t*-AUCB-treated WT. Like EpOMEs, the physiologic functions of HODEs are still being investigated [48]. 13-HODE is suggested to have an anti-inflammatory role in inflammatory diseases through its effect as a PPARγ-agonist [63–67]. Also, 13-HODE, through increasing prostacyclin (PGI_2_) biosynthesis, was involved in splenic and coronary artery relaxation in smooth muscle cells in Mongrel dogs [15]. 9-HODE, unlike 13-HODE, was described as pro-inflammatory in an experimental wound-healing model in rats [68, 69]. In contrast to 13-HODE, which can be produced from LA by the action of 12/15-LOX, 9-HODE production by LOX has not been reported in humans; rather, mouse 8-LOX metabolizes LA to 9-HODE [68]. In contrast to our data, Luria et al. reported no change in 9– and 13–HODE in sEH^–/–^compared to sEH^+/+^ [10]. This discrepancy may be explained by the difference in the source of the metabolites: we used heart perfusate samples, whereas Luria et al [10] used urinary samples. Also, the reported metabolic and functional effects of sEH^–/–^(genetic deletion of sEH) and pharmacologic inhibition of sEH are not necessarily the same [11]. Therefore, and based on our results, the increase in 13-HODE in *t*-AUCB–treated mouse heart perfusates might have played a role in enhancing CRH, whereas the role of 9-HODE is not yet known based on its, thus far, described biologic functions.

Treatment of WT mice hearts with *t*-AUCB significantly decreased prostanoid levels, including PGs and TxB_2_. PG-G_2_ and PG-H_2_, AA metabolites formed by COX isoforms (1 and 2), are converted to the 4 main bioactive PGs (PG-D_2_, PG-E_2_, PG-I_2_, PG-F_2α_) and thromboxanes (TxA_2_ and TxB_2_) [70, 71]. Most PGs have pro-inflammatory effects. For example, PG-E_2_ augments arterial dilation and increases microvascular permeability [71]. However, PG-E_2_ was found to have an anti-inflammatory role as well by up-regulating cAMP and inducing secretion of the anti-inflammatory IL-10 [72]. Similarly, PG-D_2_ attenuated inflammation in experimental models of pleuritis and colitis [71]. 6-keto-PG-F_1α_, the non-active hydrolysis product of prostacyclin (PG-I_2_), and TxB_2_, the inactive degradation product of TxA_2_ [71], were also decreased by *t*-AUCB. PG-F_2α_ is elevated in patients with chronic inflammatory diseases [71]. In support of our findings, sEH-inhibition by AUDA-BE reversed lipopolysaccharide (LPS)-induced increase in PG-D_2_, PG-E_2_, 6-keto-PG-F_1α_, and TxB_2_ [13]. Also, sEH-inhibition by AUDA-BE, through increasing the concentrations of EETs, inhibited NF-κB translocation [12]. Combination of sEH-inhibition by AUDA-BE and COX-inhibition had an additive effect and further decreased PG-D_2_ and PG-E_2_ [12]. Therefore, the associated decrease in the measured PGs could be attributed to sEH-inhibition by *t*-AUCB. However, the impact of these changes on the *t*-AUCB–enhanced CRH is not yet clear.

As mentioned earlier, EETs are generated from AA by cytochrome P450 (CYP) epoxygenase enzymes, primarily CYP2C and CYP2J. We targeted the CYP epoxygenase-EET pathway using pharmacologic inhibition of CYP-epoxygenases to further support our findings of the involvement of CYP-epoxygenases in modulating CRH. sEH-inhibition increases the level of EETs by blocking their breakdown; we investigated the effect of blocking the synthesis of EETs on CRH using a CYP epoxygenases-inhibitor. Evidently, inhibiting CYP epoxygenases would negatively impact EET synthesis and, therefore, their effects. P450 enzymes are highest in coronary arteries and in small arterioles [2]. MS-PPOH was used as an EET-synthesis inhibitor that blocked the role of EETs as mediators of insulin-mediated augmentation of skeletal muscle perfusion [73]. Our data indicate that MS-PPOH significantly decreased CRH after a brief ischemia. These findings suggest that inhibition of CYP epoxygenases by MS-PPOH decreased CRH possibly due to inhibition of EETs synthesis. Further studies to investigate the impact of MS-PPOH on oxylipin profiles are needed to understand the metabolic changes associated with the inhibition of CYP epoxygenases.

This study also showed that CRH was decreased by T0070907, a PPARγ-antagonist, whereas rosiglitazone, a PPARγ-agonist, increased CRH. Moreover, T0070907 attenuated *t*-AUCB–enhanced CRH in WT mouse hearts. Studies indicated that EET effects could be mediated by PPARγ receptors [20–23]. Liu et al. suggested that selective sEH inhibition potentiates the anti-inflammatory effect in endothelial cells by increasing the retention of EETs so that PPARγ activation is prolonged [20]. Our results suggest that PPARγ receptors are involved in modulating CRH. Also, the attenuated *t*-AUCB–enhanced CRH in WT mice (by T0070907) suggests that PPARγ may mediate CRH downstream of CYP epoxygenases-EET pathway.

In summary, the findings of this study suggest that the CYP epoxygenases-EET pathway may have a role in mediating CRH possibly through PPARγ activation, as noted by sEH-inhibition, which enhanced CRH, and CYP epoxygenases-inhibition, which decreased CRH. Also, sEH-inhibition is accompanied by changes in AA oxylipins (increased EET/DHET ratio, decreased mid-chain HETEs, and decreased prostanoids) and LA oxylipins (increased EpOME/DiHOME ratio, and increased HODEs), which might have collectively accounted for the observed enhancement of CRH. Therefore, we conclude that sEH-inhibition enhances, whereas CYP epoxygenases-inhibition attenuates CRH, PPARγ activation mediates CRH downstream of the CYP epoxygenases-EET pathway, and the changes in oxylipin profiles associated with sEH-inhibition collectively account for the enhanced CRH.
